# Identification and evaluation of PCR reference genes for host and pathogen in sugarcane*-Sporisorium scitamineum* interaction system

**DOI:** 10.1186/s12864-018-4854-z

**Published:** 2018-06-19

**Authors:** Ning Huang, Hui Ling, Feng Liu, Yachun Su, Weihua Su, Huaying Mao, Xu Zhang, Ling Wang, Rukai Chen, Youxiong Que

**Affiliations:** 1 Key Laboratory of Sugarcane Biology and Genetic Breeding, Ministry of Agriculture, Fujian Agriculture and Forestry University, Fuzhou, 350002 China; 2 Key Laboratory of Ministry of Education for Genetics, Breeding and Multiple Utilization of Crops, College of Crop Science, Fujian Agriculture and Forestry University, Fuzhou, 350002 China; 3 Guangxi Collaborative Innovation Center of Sugarcane Industry, Guangxi University, Nanning, 530005 China

**Keywords:** *Saccharum* L., Smut fungus, Quantitative real-time PCR, Reference gene

## Abstract

**Background:**

Sugarcane (*Saccharum* L. plant) is an important crop for sugar and bio-energy production around the world. Among sugarcane diseases, smut caused by *Sporisorium scitamineum* is one of the major fungal diseases causing severe losses to the sugarcane industry. The use of PCR reference genes is essential to the normalization of data on gene expression involving the sugarcane-*S. scitamineum* interaction system; however, no report that addresses criteria in selecting these reference genes has been published to date.

**Results:**

In this study, 10 sugarcane genes and eight *S. scitamineum* genes were selected as candidate PCR reference genes in the sugarcane-*S. scitamineum* interaction system. The stability and reliability of these 18 candidate genes were analyzed in smut-resistant (NCo376) and -susceptible (YC71–374) genotypes using the statistical algorithms geNorm, NormFinder, BestKeeper, and deltaCt method. Subsequently, the relative expression levels of the sugarcane chitinase I-3 gene and *S. scitamineum* chorismate mutase gene were determined to validate the applicability of these sugarcane and *S. scitamineum* PCR reference genes, respectively. We finally found that the acyl-CoA dehydrogenase gene (*ACAD*), serine/arginine repetitive matrix protein 1 gene (*SARMp1*), or their combination (*ACAD* + *SARMp1*) could be utilized as the most suitable reference genes for normalization of sugarcane gene expression in sugarcane bud tissues after *S. scitamineum* infection. Similarly, the inosine 5′-monophosphate dehydrogenase gene (*S10*), the SEC65-signal recognition particle subunit gene (*S11*), or their combination (*S10* + *S11*) were suitable for normalization of *S. scitamineum* gene expression in sugarcane bud tissues.

**Conclusions:**

The PCR reference genes *ACAD*, *SARMp1*, *S10*, and *S11* may be employed in gene transcriptional studies involving the sugarcane-*S. scitamineum* interaction system.

**Electronic supplementary material:**

The online version of this article (10.1186/s12864-018-4854-z) contains supplementary material, which is available to authorized users.

## Background

Regarding the development of technologies in biological research, omics data have led to a considerable increase in gene identification [1]. Gene expression analysis is essential in understanding the signaling and metabolic pathways that underly cellular and developmental processes [2]. Currently, real-time quantitative PCR (qRT-PCR), semi-quantitative PCR (semi-qPCR), and northern blotting are the major methods of quantifying and validating the expression of genes [3–6]. Reference genes, which are expressed stably in different organ tissues, at different developmental stages, or under specific-experimental conditions, can improve the precision of gene expression quantification by reducing experimental errors caused by RNA quality, cDNA synthesis, qPCR reactions, or other factors [7]. Previously, housekeeping genes, which were indispensable in maintaining basic metabolic activities and basic structural components, were widely used as PCR reference genes for gene expression analysis in humans [8], animals [9] and plants [10]. Furthermore, expression analysis of pathogen genes during infection and colonization in the host plant has been the main step in elucidating the biological function of pathogen genes. To reduce errors in expression quantification of pathogen genes caused by the adaptability of the pathogen, as well as nutrition and stress in host plants [11, 12], pathogen PCR reference genes have been included in investigations. However, there is growing evidence showing there is no single, universal gene that could be utilized in various experimental conditions [13], and the stability of reference genes should be validated before these are used for normalization of gene expression [14, 15].

Over the past decades, the evaluation of PCR reference genes has been reported in various plant species such as *Oryza sativa* [16], *Zea mays* [17], *Brassica juncea* [18], *Triticum aestivum* [19], and *Nicotiana tabacum* [20]. Sugarcane (*Saccharum* L.), which belongs to *Gramineae*, is an important crop for sugar and bio-energy production in more than 110 tropical and subtropical countries around the world [21]. Sugarcane PCR reference genes, which are used in the normalization of gene expression in different tissues of sugarcane varieties (glyceraldehyde-3-phosphate dehydrogenase, *GAPDH*) [22] or under different abiotic stresses (eukaryotic elongation factor 1A, *eEF1A*; cullin, *CUL*; and clathrin adaptor complex, *CAC*) [23–25], have been evaluated and reported. PCR reference genes that are identified by commonly used algorithms geNorm [26], NormFinder [27], BestKeeper [28], and the deltaCt method [8] allow reliable and accurate normalization for gene expression data [29–32]. Analysis using geNorm is based on two reference genes that would show highly identical expression pattern across different experimental biological samples [26]. NormFinder is a mathematical algorithm that estimates gene expression stability by comparing variations of gene expression in intra/inter-groups within a group or groups of bio-samples [27]. BestKeeper assesses the expression stability of genes by employing Pearson, Spearman, and Kendall Tau correlation coefficients for pair-wise correlation analysis, generating weighted indices of the candidate genes [28]. The deltaCt method [26] describes the ΔCt approach, which requires less use of specialist programs and biomaterials by comparing pairs of candidate genes [8]. With increasing demand for sugarcane production, a growing number of RNA-seq trials have been conducted to identify genes that are associated with specific biological processes such as sugar accumulation [33], fiber content [34], and stress responses [3, 4, 23–25]. Smut disease, which is caused by *Sporisorium scitamineum*, is one of the major fungal affecting sugarcane growth, often resulting in a 3%–7% reduction in sugar content [21]. Wu et al. [4] identified 2015 sugarcane differentially expressed ESTs using the Solexa sequencing technology, and Que. et al. [3] identified a total of 65,852 sugarcane unigenes by RNA sequencing during *S. scitamineum* infection. Genome sequencing has indicated that the genome of *S. scitamineum*, which is 19.63 Mb and 19.98 Mb in size, consists of about 6636 to 6693 genes, which include 527 secreted protein genes, 192 pathogenic genes, and 68 effector protein genes [35–37]. Yan et al. [38] identified 52 genes that were regulated by the b-locus by de novo RNA-sequencing of *S. scitamineum* and its resultant *SsΔMAT-1b* mutant. By comparing the transcriptome of *S. scitamineum* in sugarcane tissues and in vitro, Taniguti et al. [36] identified 125 differentially expressed genes at 5 d and 907 differentially expressed genes at 200 d. Although numerous sequences of differentially expressed genes have been isolated from sugarcane and *S. scitamineum* after infection [4, 36, 38–41], details of the underlying regulatory network remain unclear. Expression profiling of defense-related genes in sugarcane and pathogenesis-related genes in *S. scitamineum* is essential to the elucidation of the molecular basis of the sugarcane-*S. scitamineum* interaction system. To date, no report on reference gene selection for gene expression normalization in sugarcane-*S. scitamineum* interaction system has been published.

In this study, 10 sugarcane candidate PCR reference genes were obtained, including six (acyl-CoA dehydrogenase, *ACAD*; casein kinase I isoform delta-like, *CK1δ*; OTU domain-containing protein 5, *OTU5*; 12-oxophytodienoate reductase 7, *OPR7*; polyadenylate-binding protein 8, *PABP8*; serine/arginine repetitive matrix protein 1, *SARMp1*) from our previous transcriptomic data [3] and four (*GAPDH*, *eEF1A*, *CUL*, and *CAC*) from previous reports [23, 24]. In addition, eight *S. scitamineum* candidate PCR reference genes (conserved hypothetical protein, *S2*; conserved hypothetical protein, *S4*; VPS73-protein involved in vacuolar protein sorting, *S6*; synaptobrevin, *S8*; GTP-binding protein Rac1, *S9*; inosine 5`-monophosphate dehydrogenase, *S10*; SEC65-signal recognition particle subunit, *S11*; and ADP-ribosylation factor, *S12*) were selected from a *S. scitamineum* genomic-wide expression profile microarray (unpublished, Huang et al., hning2012@126.com). The expression of the 18 candidate PCR reference genes in smut-infected buds of smut-susceptible genotype YC71–374 and smut-resistant genotype NCo376 was assessed using qRT-PCR, and their stabilities were evaluated using algorithms geNorm, NormFinder, BestKeeper, and the deltaCt method. This study aimed to identify suitable PCR reference genes for accurate normalization and quantification of gene expression levels of the host and pathogen in a sugarcane-*S. scitamineum* interaction system.

## Methods

### Plant materials and treatment

The smut-resistant genotype NCo376 and the -susceptible genotype YC71–374 were provided by the Key Laboratory of Sugarcane Biology and Genetic Breeding, Ministry of Agriculture, Fujian Agriculture and Forestry University. The disease-free sugarcane materials were collected and cut into single bud canes, immersed in 5 g/L carbendazim (Shanghai Huanong Chemical Co. Ltd., Shanghai, China) for 48 h, and then transferred into a 50 °C water bath for 2 h. The buds were embedded in sterile nutritional soil in an incubator with 28 + 0.5 °C, 3000 lx, and 16 h light/8 h dark conditions. After incubation for 7 d to 10 d, the buds grew to a length of 1~ 2 cm and were divided into two groups, namely, the control group and experimental group. And then these two groups were injected with 0.5 μL sterile water (0.01% *V*/V, Tween-20/water) and 0.5 μL of a *S. scitamineum* spores suspension (density: 5 × 10^6^/mL, 0.01% *v*/v, Tween 20/water) respectively, and placed into the same incubator. Both groups consisted of three biological replicates. Each sample included five sugarcane buds, which were excised for RNA extraction at 0 d, 3 d, and 7 d after injection. All samples were immediately frozen in liquid nitrogen and stored at − 80 °C until use.

### RNA isolation and cDNA synthesis

RNA was isolated using TRIzol (Invitrogen, Shanghai, China) and assessed in quantity and quality using a multifunction microplate reader Synergy H1 (Bio-Tek, Winooski, VT, USA) and 1.5% agarose gel electrophoresis. Prior to cDNA synthesis, any contaminating genomic DNA in the total RNA samples was removed using RNase-free DNase I (Promega, WI, USA). cDNA was synthesized using a PrimeScript RT kit (Perfect for Real Time) (TaKaRa Biotech., Dalian, China) following the manufacturer’s recommendations, resolved using 1.5% agarose gel electrophoresis, and then stored at − 20 °C. To exclude the genomic DNA contaminating cDNA samples, all the cDNA samples were detected by the SD7R primers (ACTTACGAGCACCTCAGGGA/AGAGTCCGAAGCCGAAGAT) before use it as qRT-PCR template, which could achieve two fragments in genomic DNA samples and one fragment in cDNA samples [42].

### Identification of candidate PCR reference genes and primer design

*GAPDH*, *eEF1A*, *CUL*, and *CAC* were chosen as sugarcane candidate PCR reference genes in the present study based on previous reports [23, 24]. The other six sugarcane candidate PCR reference genes (*ACAD*, *CK1δ*, *OTU5*, *OPR7*, *PABP8*, and *SARMp1*), which were expressed stably at higher levels than that of *GAPDH* in the transcriptomic data of sugarcane under *S. scitamineum* stress [3], were also chosen. Similarly, based on the expression profile of *S. scitamineum* genes in the genome-wide expression profile microarray (unpublished, Huang et al., hning2012@126.com), eight *S. scitamineum* genes (*S2*, *S4*, *S6*, *S8*, *S9*, *S10*, *S11*, and *S12*) with high and stable expression levels were selected as *S. scitamineum* candidate PCR reference genes. Furthermore, except for *eEF1A*, *CUL*, and *CAC* [23], the qRT-PCR primers of all the other candidate PCR reference genes were designed using Primer-BLAST (https://www.ncbi.nlm.nih.gov/tools/primer-blast/) and presented in Table 1.

**Table 1:** Primers of candidate PCR reference genes

Category	Gene Name	Sense Primer/Anti-sense Primer (5′-3′)
Sugarcane	Acyl-CoA dehydrogenase family member 10, *ACAD*	CGTGGCATGGATCTGATGGT/AGCCTGCTCCAGTTCAATCC
Sugarcane	Casein kinase I isoform delta-like, *CK1δ*	TCAAGGGCTACCTCCCTCTC/GCATTCTTCCCTTCCGCTCT
Sugarcane	OTU domain-containing protein 5, *OTU5*	TGGTGCAGAGCCCATTAACA/GCCTTCACCTGGTCCCTATC
Sugarcane	12-oxophytodienoate reductase 7, *OPR7*	TGTTCATCGCTAACCCGGAC/TTAGGCTGGCCAAGGAATGG
Sugarcane	Polyadenylate-binding protein 8, *PABp8*	TTGGGACTCTGACTTCTGCC/CCAGTGACCTTTGCTGCTTG
Sugarcane	Serine/arginine repetitive matrix protein 1, *SARMp1*	TGGACTTGGTCAGTTGGAAACA/TGTTCCTGAAGCCTATGTTGCT
Sugarcane	Glyceraldehyde-3-phosphate dehydrogenase, *GAPDH*	AGGACTCCAAGACCCTCCTC/CTTCTTGGCACCACCCTTCA
Sugarcane	Eukaryotic elongation factor 1A, *eEF1A*^a^	TTTCACACTTGGAGTGAAGCAGAT/GACTTCCTTCACAATCTCATCATAA
Sugarcane	Cullin, *CUL*^a^	TGCTGAATGTGTTGAGCAGC/TTGTCGCGCTCCAAGTAGTC
Sugarcane	Clathrin adaptor complex, *CAC*^a^	ACAACGTCAGGCAAAGCAAA/AGATCAACTCCACCTCTGCG
*Sporisorium scitamineum*	Conserved hypothetical protein, *S2*	ACCTCGAGCAGCAACAGTG/ACCACAATCCAGAACTCGACG
*Sporisorium scitamineum*	Conserved hypothetical protein, *S4*	GACGGTGCCCAAGAACAGAG/CTGTGAGCTTCCAATTCCGC
*Sporisorium scitamineum*	VPS73-protein involved in vacuolar protein sorting, *S6*	AAAACCTAATGGTGGGCTCGG/GACCCAACCCGAACGAGAAC
*Sporisorium scitamineum*	Synaptobrevin, *S8*	TGCACAAGACCATCGACTCA/CGAGTTTTGCTTCTTGGCTGT
*Sporisorium scitamineum*	GTP binding protein Rac1, *S9*	CACGTGATATCCATGCGAACAAG/GAGAATGGTGCAGTTGTTCTTCT
*Sporisorium scitamineum*	Inosine 5`-monophosphate dehydrogenase, *S10*	CGTTGCAGGACATGGGTGTG/TTCTCGTAGCTGTGCAGACCA
*Sporisorium scitamineum*	SEC65-signal recognition particle subunit, *S11*	GAATGCTTGGAGGCATGGGG/GCGGGTTCATAGGGTCCTTC
*Sporisorium scitamineum*	ADP-ribosylation factor, *S12*	AACGACCGAGAGCGTGTTTC/AGCTTGTCCGTAATCTCGGC

Note: ^a^Ling et al., 2014

### qRT-PCR and data analysis

qRT-PCR was prepared using the SYBRGreen Universal Master Mix kit (Roche, NY, USA) and consisted of a cDNA template (equivalent to 10 ng of RNA), primers, and ddH_2_O. Each qPCR reaction contained three technical replicates and used ddH_2_O as a blank control. The qRT-PCR amplification program was as follows: 50 °C for 2 min, 95 °C for 10 min, followed by 40 cycles of 95 °C for 15 s, 60 °C for 1 min, which were the default parameters of the ABI 7500 FAST Real-Time PCR System. Using a series of gradient-diluted cDNA samples as the template, the Ct value of each primer pair was calculated after qRT-PCR analysis and was used to generate a standard curve, following the amplification efficiency of each primer pair [23].

In the present study, 36 cDNA samples (0 d, 3 d, and 7 d samples in the experimental group and the control group, including three biological replicates) were used to assess the expression of 10 sugarcane candidate PCR reference genes. For undetection or inexistence of *S. scitamineum* cells in the 0 d samples in the experimental and the control groups, only 3 d and 7 d samples in the experimental group (12 cDNA samples, each sample includes three biological replicates) were used for assessing the eight *S. scitamineum* candidate PCR reference genes. The cycle threshold (Ct) values of all samples were used to calculate the mean value, standard deviation, and covariation (CV, CV = Standard deviation/Average Ct value × 100%) value. The mean Ct values were ∆Ct-transformed and then directly imported into geNorm [26] and NormFinder [27], following the manuals’ instructions. Analysis of the stability coefficient of the candidate PCR reference genes on BestKeeper [28] and use of the deltaCt method [8] was as previously described. Pearson correlation coefficients (r values) were analyzed with the software SAS S21.0 using the stability values (SV) generated by geNorm, NormFinder, deltaCt, and BestKeeper [24]. The algorithms with generally similar results were used to generate the comprehensive stability values [24]. The relative SVs (Relative SV = (SV of rank N)/(SV of Rank 1), *N* = 1~ 10) were achieved based on the SVs from the algorithms and the deltaCt method. The comprehensive SV (CSV) was computed from the geometrical mean (GM) of the relative SVs of each candidate PCR reference gene. The best combination of PCR reference genes was recommended by the geNorm [26]. To verify the reliability of the selected PCR reference genes, the expression of sugarcane chitinase I-3 (*ScChi I-3*) [43] and *S. scitamineum* chorismate mutase (*SsCMU*) [44] was normalized using the selected candidates, including the best candidate PCR reference gene, the most variable candidate PCR reference gene, and the optimal combination candidate PCR reference genes, respectively. The relative expression of *ScChi I -3* and *SsCMU* was calculated with the formula previously described by Pfaffl et al. [45], which are both implemented in OriginPro 9.2.

## Results

### Identification of candidate PCR reference genes

Because the CV value is an indicator of the degree of discretization in a group, the lower the CV value, the less variation among groups. In transcriptomic data shown in Table 2 [3], except for *CAC*, the CV values of *GAPDH*, *eEF1A*, and *CUL* were 2.52%, 12.59%, and 5.22%. The CV values of *ACAD*, *CK1δ*, *OTU5*, *OPR7*, *PABP8*, and *SARMp1* were between 2.65% and 3.88%, and their expression was higher than that of *GAPDH* (Table 2). However, the expression levels of the eight *S. scitamineum* genes, *S2*, *S4*, *S6*, *S8*, *S9*, *S10*, *S11*, and *S12* were also relatively high; however, these showed slight variations (1.61%~ 4.31%) in the genomic-wide expression profile microarray (Table 3). The above 10 sugarcane genes and eight *S. scitamineum* genes were then selected as candidate PCR reference genes (Table 1) and further evaluated.

**Table 2:** The expression analysis of ten sugarcane candidate PCR reference genes (CRG) based on the RPKM value from RNA-seq data

CRG	YC05–179				ROC22				mean	SD	CV
	CK-24 h	J-24 h	J-48 h	J-120 h	CK-24 h	J-24 h	J-48 h	J-120 h			
*ACAD*	37.82	37.89	34.78	37.11	37.01	34.56	35.16	36.61	36.37	1.35	3.70%
*CK1δ*	91.73	89.52	94.76	92.68	94.37	87.24	93.32	98.05	92.71	3.32	3.58%
*OTU5*	69.95	72.46	71.13	77.11	71.21	69.04	75.94	71.98	72.35	2.81	3.88%
*OPR7*	51.73	52.14	55.65	53.10	48.94	52.02	51.81	50.56	51.99	1.93	3.72%
*PABp8*	137.92	135.44	131.73	124.45	138.25	131.10	138.93	135.35	134.15	4.87	3.63%
*SARMp1*	31.50	32.97	31.20	32.83	32.69	31.46	31.33	30.79	31.85	0.85	2.65%
*GAPDH*	30.31	30.21	29.81	29.19	28.36	28.71	30.26	29.30	29.52	0.74	2.52%
*eEF1A*	2520.172	2233.869	2788.05	1980.246	2260.422	1920.52	2271.531	2100.286	2259.387	284.4435	12.59%
*CUL*	107.9021	105.826	101.8729	111.1481	117.8406	113.4682	118.8913	111.88	111.1037	5.800734	5.22%
*CAC*	–	–	–	–	–	–	–	–	–	–	–

Note: YC05–179 and ROC22, sugarcane genotype YC05–179 and ROC22; *CRG* candidate PCR reference gene, *RPKM* reads per kilo bases per million reads, *CK/J-24 h, −48 h and − 120 h* the data from the control/treated (CK/J) sample for CK/J-24 h, 48 h, 120 h incubation (Que et al. 2014), *SD* standard deviation, *CV* covariation, The *CAC* gene was not found in the RNA-seq data

**Table 3:** The normalized signal of eight *S. scitamineum* candidate PCR reference genes (CRG) in each sample

CRG	YC71–374												NCo376												mean	SD	CV
	0 d			3 d			5 d			7 d			0 d			3 d			5 d			7 d					
	J1	J2	J3	J1	J2	J3	J1	J2	J3	J1	J2	J3	J1	J2	J3	J1	J2	J3	J1	J2	J3	J1	J2	J3			
*S2*	10.0	10.1	10.4	10.2	10.1	10.0	10.3	10.2	10.4	10.6	10.3	10.5	10.2	10.3	10.4	10.5	10.2	10.5	10.4	10.4	10.3	10.3	10.3	10.4	10.0	0.2	1.61%
*S4*	7.7	7.8	7.9	7.8	7.8	7.5	7.9	7.9	8.1	8.1	7.7	7.8	7.9	8	7.9	8.0	8.0	8.3	7.8	7.9	7.8	8.0	7.6	8.2	7.9	0.2	2.22%
*S6*	8.1	8.1	8.3	8.1	8.2	7.9	8.0	8.0	8.2	8.2	8.1	8.2	8.5	8.4	8.4	8.4	8.1	8.4	7.7	8.3	8.0	8.3	7.9	8.6	8.2	0.2	2.58%
*S8*	10.2	9.9	10	10.2	10.6	10.3	10.4	10.2	10.6	10.3	10.0	10.4	10.9	10.7	10.6	10.6	10.5	10.8	9.3	10.7	10	10.4	9.7	10.9	10.0	0.4	3.68%
*S9*	10.9	11.1	11	11.0	11.3	10.7	10.4	10.9	11.1	11.1	11.1	10.8	11.1	11.1	11.2	10.9	11.0	11.3	9.9	10.8	10.4	10.8	9.8	10.1	11.0	0.4	3.88%
*S10*	11.2	11.3	11.2	11.5	11.8	11.3	11.7	11.3	12.8	11.9	12.4	11.6	10.9	11.3	11.1	11	11.2	11.1	11.7	11.5	11.4	11.2	10.9	11	11.0	0.5	3.99%
*S11*	7.2	7.5	7.6	7.5	7.4	7.3	7.8	7.5	8.2	7.7	8.4	7.7	7	7.6	7.6	7.5	7.4	7.2	7.9	7.6	7.5	7.3	7.3	7.4	7.5	0.3	4.05%
*S12*	7.8	8.2	8.4	8.2	7.9	7.5	8.4	8.5	8.5	7.8	8	8.1	8.0	8.0	8.1	8.5	7.9	8.3	8.0	8.5	7.2	7.8	7.6	8.6	8.1	0.3	4.31%

Note: YC71–374 and NCo376, sugarcane genotypes YC71–374 and NCo376; *CRG* candidate PCR reference gene, *J1~J3* the bio-replicate sample 1~ 3, *SD* standard deviation, *CV* covariation

### Quality evaluation of the primers of the candidate PCR reference genes

Following the MIQE guidline [46], the melting curve of each the primer pair was analyzed, and the primer pairs of all these 18 candidate PCR reference genes with high amplifying-specificity were selected and used in the subsequent qRT-PCR analysis (Additional file 1: Figure S1 and Additional file 2: Figure S2). Table 4 shows that the regression coefficients of all the primer pairs range from 0.990 to 0.999, indicating that the amplification efficiency of these primer pairs is accurate and reasonable. The amplification efficiency of the candidate PCR reference genes was between 0.95 and 1.06, and the amplicon length was between 102 bp and 186 bp (Table 4).

**Table 4:** The length of qRT-PCR amplicon and the PCR efficiency of 18 candidates PCR reference genes (CRG)

Category	CRG	Amplicon length (bp)	E (%)	R^2^
Sugarcane	*ACAD*	120	1.01	0.995
Sugarcane	*CK1δ*	153	1.01	0.993
Sugarcane	*OTU5*	186	0.99	0.992
Sugarcane	*OPR7*	133	1.05	0.992
Sugarcane	*PABP8*	121	1.03	0.993
Sugarcane	*SARMp1*	102	1.01	0.999
Sugarcane	*GAPDH*	170	1.04	0.990
Sugarcane	*eEF1A* ^a^	103	0.97	0.999
Sugarcane	*CUL* ^a^	105	1.06	0.999
Sugarcane	*CAC* ^a^	112	1.00	0.999
*Sporisorium scitamineum*	*S2*	118	1.04	0.999
*Sporisorium scitamineum*	*S4*	130	0.99	0.990
*Sporisorium scitamineum*	*S6*	138	0.96	0.995
*Sporisorium scitamineum*	*S8*	122	0.97	0.992
*Sporisorium scitamineum*	*S9*	143	1.04	0.993
*Sporisorium scitamineum*	*S10*	133	1.00	0.993
*Sporisorium scitamineum*	*S11*	102	1.00	0.995
*Sporisorium scitamineum*	S12	146	0.97	0.996

Note: ^a^, Ling et al. 2014

### Expression analysis of candidate PCR reference genes

**Fig. 1:**
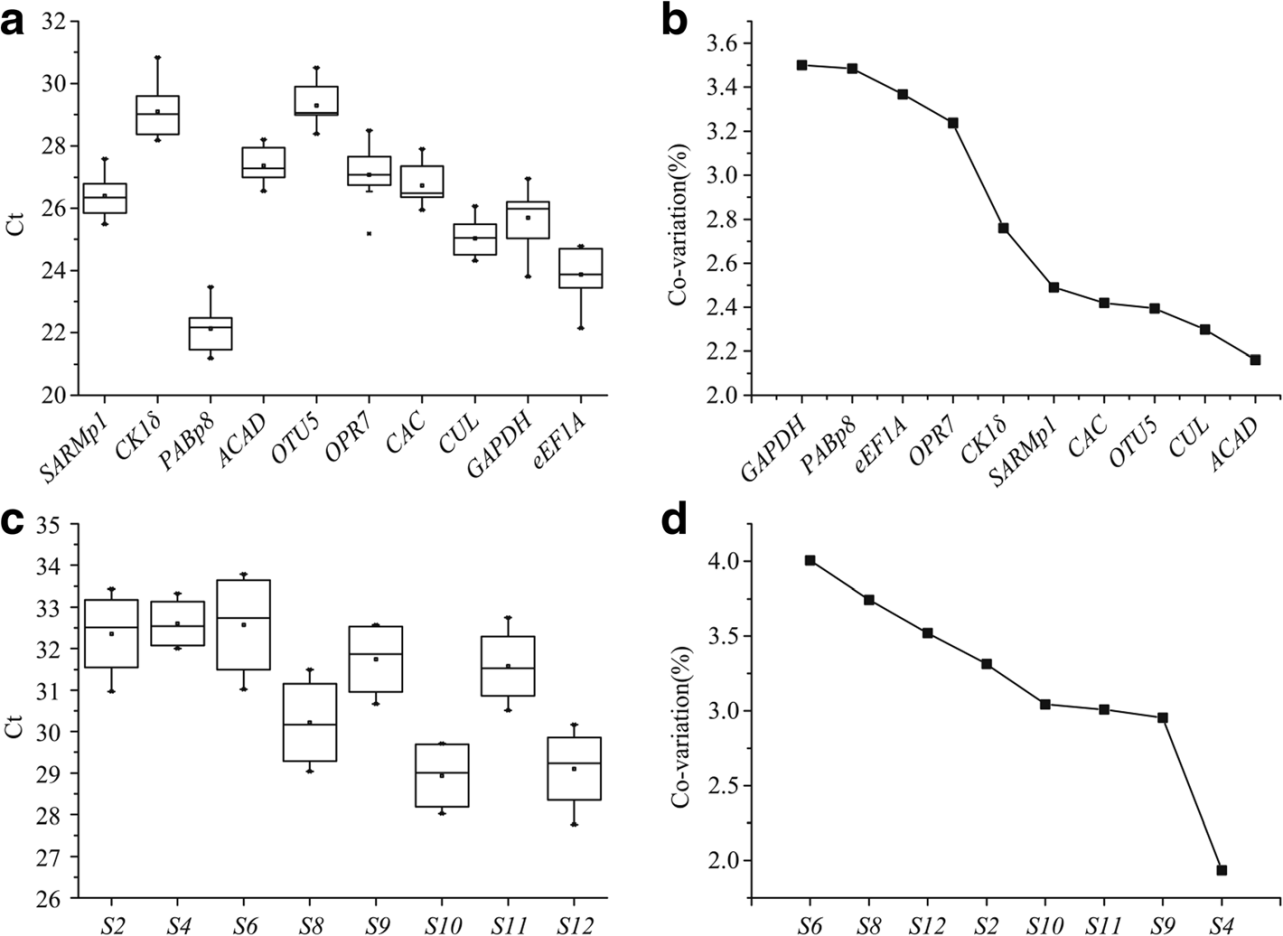
Cycle threshold (Ct) values and variations in the expression of candidate PCR reference genes. **a**, the mean Ct value of sugarcane candidate PCR reference genes; **b**, the expression covariation (CV) of sugarcane candidate PCR reference genes; **c**, the mean Ct value of *S. scitamineum* candidate PCR reference genes; **d**, the expression CV of Ct value of *S. scitamineum* candidate PCR reference genes

The expression of 10 sugarcane candidate PCR reference genes in smut spores/water-injected sugarcane (0 d, 3 d, and 7 d) and eight *S. scitamineum* candidate PCR reference genes in *S. scitamineum* spores-injected sugarcane (3 d and 7 d) were analyzed by qRT-PCR. The results showed that the Ct value of the 10 sugarcane candidates were within the range of 22.13–29.30 (Fig. 1a). The *PABp8* gene showed the highest expression level, whereas *OTU5* exhibited the lowest. The Ct value of the eight *S. scitamineum* candidate PCR reference genes was within the range of 28.94–32.61 (Fig. 1c). *S4* showed the highest expression, and *S10* exhibited the lowest expression. The CV value of the sugarcane candidates ranged from 2.16 to 3.50% (Fig. 1b), whereas that of the *S. scitamineum* candidates ranged from 1.93 to 4.00% (Fig. 1d). In terms of CV values, *ACAD*, *CUL*, *OTU5*, *CAC*, and *SARMp1* showed less variations than the remaining five sugarcane candidates (*GAPDH*, *eEF1A*, *CK1δ*, *OPR7*, and *PABP8*) with smut fungus infection, whereas *GAPDH* exhibited the highest variability (Fig. 1b). Similarly, the expression of *S4*, *S9*, *S11*, and *S10* was less variable than the remaining four *S. scitamineum* candidates (*S2*, *S6*, *S8*, and *S12*), and *S6* was the most variable *S. scitamineum* candidate PCR reference gene in *S. scitamineum*-infected sugarcane bud tissues (Fig. 1d).

### Stability analysis of candidate PCR reference genes

The algorithms geNorm, NormFinder, BestKeeper and deltaCt were used to analyze gene stability in sugarcane bud samples based on their Ct values, and the results were assessed using the Pearson correlation coefficient. Table 5 shows that different algorithms could generate significantly consistent results in stability evaluation of candidate PCR reference genes, especially geNorm, NormFinder, and deltaCt in sugarcane candidates and geNorm and NormFinder in *S. scitamineum* candidates. For its negative correlation with geNorm, NormFinder, and deltaCt, the stability value from BestKeeper was not included in the evaluation of the *S. scitamineum* candidate PCR reference genes in the present study. Tables 6 and 7 show that the genes with higher stability have the smaller stability values (SVs). In terms of the ranking of the sugarcane candidate PCR reference genes, the results generated using geNorm and NormFinder were nearly the same, and the top seven genes were *ACAD* > *SARMp1* > *CK1δ* > *CAC* > *OTU5* > *PABP8* > *CUL*. The stability order (from stable to unstable) of the sugarcane candidate PCR reference genes derived from BestKeeper was *SARMp1*, *ACAD*, *CAC*, *OTU5*, *CUL*, *PABP8*, *eEF1A*, *GAPDH*, *CK1δ*, and *OPR7* (Table 6). Except for the order of *CAC*/*CUL* and *CK1δ*/*GAPDH* being reverse, the rank of candidates generated by BestKeeper and deltaCt was generally similar. The ranking order of the *S. scitamineum* candidate PCR reference genes from geNorm and NormFinder was also the same. Among the eight *S. scitamineum* candidates, *S10* and *S11* were the two most stable genes, whereas *S6* was the most variable (Table 7). DeltaCt analysis showed that the stability of *S9* was higher than the remaining seven *S. scitamineum* candidate PCR reference genes, followed by *S10*, *S12*, and *S8* (Table 7), whereas *S2* was the most unstable (Table 7).

**Table 5:** Correlation of the stability value of reference gene based on four statistical algorithms

Algorithms	Correlation	
	Sugarcane	*Sporisorium scitamineum*
geNorm vs. NormFinder	0.964**	0.993**
geNorm vs. deltaCt	0.694*	0.376
geNorm vs. BestKeeper	0.594	−0.696
NormFinder vs. deltaCt	0.595	0.303
NormFinder vs. BestKeeper	0.425	−0.700
deltaCt vs. BestKeeper	0.927**	−0.067

Note: *, significant difference (*p* < 0.05); **, significant difference (*p* < 0.01)

**Table 6:** The relative stability value (RSV) of ten sugarcane candidate PCR reference genes

	geNorm		NormFinder		BestKeeper		DeltaCt		Comp. Rank	
	Gene	RSV	Gene	RSV	Gene	RSV	Gene	RSV	Gene	CSV
1	*ACAD*	1.00	*ACAD*	1.00	*SARMp1*	1.00	*SARMP1*	1.00	*ACAD*	1.01
2	*SARMP1*	1.00	*SARMP1*	1.08	*ACAD*	1.02	*ACAD*	1.01	*SARMp1*	1.02
3	*CK1δ*	1.74	*CK1δ*	2.00	*CAC*	1.03	*CUL*	1.06	*CAC*	1.53
4	*CAC*	2.13	*CAC*	2.36	*OTU5*	1.05	*OTU5*	1.06	*OTU5*	1.69
5	*OTU5*	2.53	*OTU5*	2.89	*CUL*	1.05	*CAC*	1.06	*CK1δ*	1.86
6	*PABP8*	3.41	*PABP8*	3.14	*PABP8*	1.08	*PABP8*	1.21	*PABP8*	1.93
7	*CUL*	4.54	*CUL*	4.11	*eEF1A*	1.23	*eEF1A*	1.35	*CUL*	2.13
8	*eEF1A*	5.49	*OPR7*	5.13	*GAPDH*	1.67	*CK1δ*	1.68	*eEF1A*	2.66
9	*GAPDH*	6.52	*eEF1A*	5.48	*CK1δ*	2.04	*GAPDH*	1.70	*GAPDH*	3.23
10	*OPR7*	7.31	*GAPDH*	5.85	*OPR7*	3.24	*OPR7*	2.02	*OPR7*	3.96

Note: *SV* stability value, *Comp. Rank* comprehensive rank, *RSV* relative SV, *CSV* comprehensive SV

**Table 7:** The relative stability value (RSV) of *S. scitamineum* candidate PCR reference genes

	geNorm		NormFinder		DeltaCt		Comp. Rank	
	Gene	RSV	Gene	RSV	Gene	RSV	Gene	CSV
1	*S10*	1.00	*S10*	1.00	*S9*	1.00	*S10*	1.01
2	*S11*	1.00	*S11*	1.00	*S10*	1.02	*S11*	1.12
3	*S4*	2.69	*S4*	4.96	*S12*	1.39	*S4*	2.68
4	*S12*	15.34	*S12*	53.21	*S8*	1.40	*S12*	10.43
5	*S8*	21.48	*S8*	98.47	*S11*	1.41	*S8*	14.36
6	*S9*	31.63	*S9*	166.06	*S4*	1.45	*S9*	17.38
7	*S2*	37.00	*S2*	169.24	*S6*	1.47	*S2*	23.71
8	*S6*	44.43	*S6*	231.21	*S2*	2.13	*S6*	24.72

Note: *SV* stability value, *Comp. Rank* comprehensive rank, *RSV* relative SV, *CSV* comprehensive SV

Comprehensive analysis using geNorm, NormFinder, BestKeeper, and deltaCt indicated that the two most stable sugarcane candidate PCR reference genes were *ACAD* and *SARMp1*, followed by *CAC*, whereas the most variable reference gene was *GAPDH*. Similarly, based on the results of geNorm, NormFinder, and deltaCt, *S10* and *S11* are the two most stable *S. scitamineum* candidate PCR reference genes, followed by *S4*, and *S6* is the most unstable.

### Selection of the optimal combination of PCR reference genes

**Fig. 2:**
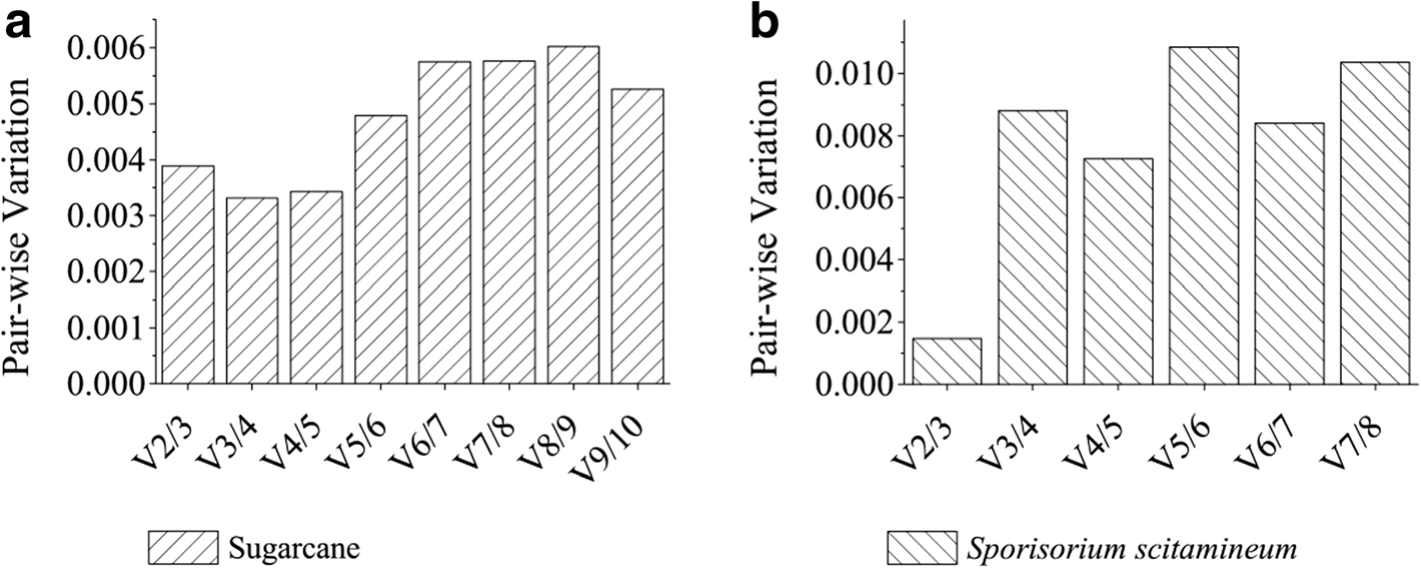
Determination of the optimal number of sugarcane (**a**) and *S. scitamineum * (**b**) reference genes for normalization by pairwise variation. Pairwise variation (Vn/Vn + 1) was analyzed between normalization factors NFn and NFn + 1 by geNorm algorithm to determine (V < 0.15) the optimal number of reference genes

Concurrently, the pairwise variation (Vn/Vn + 1) of candidate PCR reference gene combination was analyzed using geNorm, which based on the normalized Ct value [26]. The pairwise variations could reflect the variations among different gene groups, which comprised various genes. Vandesompele et al. [25] emphasized that if the pairwise variation is < 0.15, then the combination of the top n genes was more stable than the combination of top n + 1 genes [26]. Figure 2 shows that all pairwise variations of sugarcane and *S. scitamineum* genes are < 0.15. Comparisons of the pairwise variations between different gene combinations indicated minimal differences among the V2/3, V3/4, and V4/5 of the sugarcane candidate PCR reference genes, and V2/3 was the smallest among all combinations of *S. scitamineum* candidate PCR reference genes. As using fewer PCR reference genes to achieve the most reasonable results is cost-effective, “*ACAD* + *SARMp1*” and “*S10* + *S11*” are regarded as the best combinations of sugarcane and *S. scitamineum* candidate PCR reference genes, respectively.

### Validation analysis of PCR reference genes

Based on the results of our analyses, the top two stable sugarcane/*S. scitamineum* reference genes (*ACAD* and *SARMp1*/*S10* and *S11*), a moderately stable gene (*CAC*/*S4*), the most variable candidate PCR reference gene (*GAPDH*/*S6*), and the optimal combination of PCR reference genes (*ACAD* + *SARMp1*/*S10* + *S11*) were selected for further validation. Here, sugarcane *ScChi I-3* gene and *S. scitamineum SsCMU* gene were used to verify the reasonability and feasibility of the above selected genes.

**Fig. 3:**
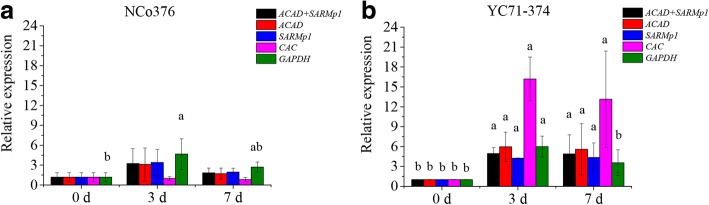
The relative expression of sugarcane chitinase I-3 gene in NCo376 (**a**) and YC71–374 (**b**) bud tissue under *S. scitamineum* infection. The a and b represent significant differences between the normalization of different reference genes or gene combinations

Figure 3a shows that the *ScChi I-3* gene was upregulated and exhibited a similar expression pattern when it was normalized with *ACAD*, *SARMp1*, and “*ACAD* + *SARMp1*” in the *S. scitamineum*-infected buds tissue samples of NCo376. The expression level of *ScChi I-3*, which was normalized with *GAPDH*, was also upregulated but was higher than that of *ACAD*, *SARMp1*, and “*ACAD* + *SARMp1*”, respectively. On the contrary, the expression of *ScChi I-3* was downregulated when normalized with *CAC*. Similarly, the normalization of *ScChi I-3* expression with reference gene “*ACAD* + *SARMp1*”, *ACAD*, *SARMp1*, and *GAPDH* was similar in the infected buds tissues of YC71–374 (Fig. 3b), whereas the *ScChi I-3* expression level with *CAC* as reference was relatively higher.

**Fig. 4:**
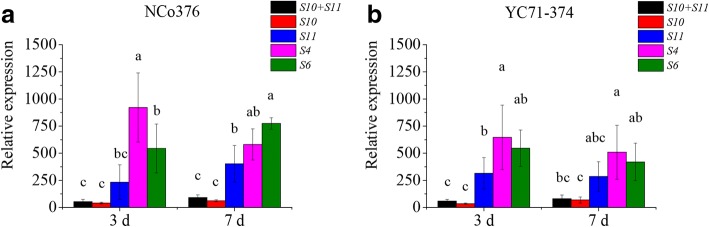
The relative expression of *S. scitamineum* chorismate mutase gene in *S. scitamineum*-infested sugarcane NCo376 (**a**) and YC71–374 (**b**) bud tissues. a, b, and c represent significant differences in normalization of different reference genes or gene combinations

The expression level of the *SsCMU* gene could not be detected in *S. scitamineum*-infected sugarcane buds at 0 d. Figure 4 (0 d was excluded in the analysis) shows that the expression of *SsCMU* gradually increased with *S. scitamineum* infestation in sugarcane tissues when the data were normalized using the two best candidate PCR reference genes (*S10* and *S11*) and their combination. Otherwise, *SsCMU* expression with *S4* or *S6* normalization was higher and differed from that with normalization using *S10*, *S11*, or their combination (*p* < 0.05) (Fig. 4). Moreover, the expression of *SsCMU* among the three bio-replicates, which was normalized with *S4* or *S6*, was relatively more variable (with greater ranges) (Fig. 4).

## Discussion

Quantification of defense response-related gene expression is an important method for elucidating the molecular mechanisms of plant-pathogen and plant-environmental factor interactions [47]. Compared to Northern blotting, ribonuclease protection assay (RPA), semi-qPCR, molecular in situ hybridization, and cDNA microarray, the combination of qRT-PCR and data normalization using PCR reference genes is a more rapid, convenient, and reliable way of assessing gene transcriptional levels [46, 48]. It has eventually become one of the most indispensable means for assessing gene expression in animals and plants [49]. The ideal reference gene should have stable expression levels in bio-samples, encodes a functional protein, and is transcriptionally abundant (with a Ct ranged within 15 to 30) [50]. It should also reflect variations in RNA extraction, cDNA synthesis, and PCR amplification [50]. Ten sugarcane genes (*GAPDH*, *eEF1A*, *CUL*, *CAC*, *ACAD*, *CK1δ*, *OTU5*, *OPR7*, *PABP8*, and *SARMp1*) and eight *S. scitamineum* genes (*S2*, *S4*, *S6*, *S8*, *S9*, *S10*, *S11*, and *S12*) were selected as candidate PCR reference genes for stability evaluation. According to the MIQE criteria [46], qRT-PCR primer pairs with amplification efficiency within the range of 0.95–1.05 were selected for quantitative expression profiling. Pearson correlation analysis showed that two algorithms, namely, geNorm and NormFinder, and the deltaCt method shared more similar ranking results candidate in PCR reference genes, whereas less similar findings were generated between BestKeeper and the algorithms geNorm, NormFinder or the deltaCt method, especially in the smut candidate PCR reference genes. Finally, three algorithms, namely, geNorm, NormFinder BestKeeper, and the deltaCt method were used in the analysis of sugarcane candidate PCR reference genes, while two algorithms, geNorm, NormFinder, and deltaCt method were used in the analysis of smut candidate PCR reference genes.

### Selection of sugarcane PCR reference genes

On the basis of reference gene selection criteria [50], the Ct values of the 18 candidate PCR reference genes were within the range 15–30, except for *CK1δ* and *OTU5* (Ct value> 30) (Fig. 1a). Previous studies have shown that the sugarcane *GAPDH* and *eEF1A* genes were stable and were deemed suitable for use as PCR reference genes under osmotic, drought, or hormonal stress [23–25], but were variable during *S. scitamineum* infection in sugarcane buds in the present study (Fig. 1b). Similarly, the expression of *GAPDH* in plant tissues varied under biotic stress such as in virus-infected *N. benthamiana* [51], rust-infected *Vigna angularis* [52], and fungi-infected *Withania somnifera* [53]. Xiao et al. [50] reported that the expression level of *eEF1A* in Chinese cabbage was altered by fungal infection [54]. This indicates that the expression of *GAPDH* and *eEF1A* in plants is sensitive to biotic stress. The transcriptomic data and the present data from geNorm, NormFinder, BestKeeper, and deltaCt method indicated that *ACAD* and *SARMp1* are the two most stable genes among the 10 sugarcane candidates. The ACAD protein is involved in the electron transport chain and is one of the most basic enzymatic components in peroxisomes, which is a basic organelle of eukaryotic cells [55]. The findings of the present study suggest that *ACAD* is suitable for use as a reference gene for normalization of data on differentially expressed genes that are related to sugarcane-*S. scitamineum* interactions, although *ACAD* has not been employed as a reference gene prior to this study. Similarly, the SARMp1 protein is a member of serine/arginine-rich protein family, which participates in the splicing of mRNA precursors [56, 57]. Compared to *GAPDH*, *EF1A*, *CAC*, and *CUL* [23, 24], both *ACAD* and *SARMp1* are the basic components for cellular structures and may also serve as PCR reference genes in sugarcane. Several previous studies have proven that the gene combination recommended by geNorm is the most suitable internal reference for gene expression [58–60]. In the present study, the results from geNorm support that *ACAD* and *SARMp1* could be used as a combination for normalization of data on gene expression.

Based on all the above results, the top two stable genes *ACAD* and *SARMp1* and the moderately stable gene *CAC* plus the unstable gene *GAPDH* were selected for further validation in *S. scitamineum*-infected NCo376 and YC71–374 bud samples. The results finally showed that the transcript profile of *ScChi I -3* that was normalized using *CAC* and *GAPDH* was significantly different from those by *ACAD*, *SARMp1* and “*ACAD* + *SARMp1*”, and was more obviously changeable. In addition, *ACAD* and “*ACAD* + *SARMp1*” generated the most similar normalization results in both smut-susceptible and -resistant genotypes in the present study. In sum, based on the evaluation of four algorithms and qRT-PCR validation, *ACAD* or “*ACAD* + *SARMp1*” are deemed the most suitable PCR reference genes for transcriptional quantification in *S. scitamineum*-infected sugarcane buds.

### Selection of *S. scitamineum* PCR reference genes

With the completion of the *S. scitamineum* genome sequencing, nearly 6636–6693 genes have been identified, which consist of 527 secreted protein genes, 192 pathogenic genes, and 68 effector proteins [35–37]. Some *S. scitamineum*-specific genes are expressed as part of the immune response of sugarcane cells to infection [36]. Prior to the identification of the function of these pathogenesis-related *S. scitamineum* genes, it is essential to know which gene is expressed differentially during infection. Internal PCR reference genes could enhance the reliability of identification of differentially expressed genes using qRT-PCR [3]. However, no report on the appropriate *S. scitamineum* PCR reference genes has been published to date.

Based on the data from a genomic-wide expression profile microarray (unpublished), eight *S. scitamineum* candidate PCR reference genes were selected for further stability evaluation using qRT-PCR and different algorithms. The results showed that the accumulation of *S10* (mean Ct = 28.94) and *S12* (mean Ct = 29.10) was within the Ct range of 15 to 30. During the detection of the *S. scitamineum* gene in sugarcane bud tissues, the transcriptional abundance of *S. scitamineum* genes is predictably lower than that of host genes, resulting in the Ct value of six *S. scitamineum* genes > 30 Ct. In other words, if the Ct value of the pathogen gene is > 30, then it does not mean that the expression of the *S. scitamineum* gene is low. Vieira et al. found that the Ct values of the *Colletotrichum kahawae* genes in the tissues of Arabica coffee hypocotyls were higher than in the medium [48]. Therefore, *S2*, *S4*, *S6*, *S8*, *S9*, and *S11* in this study could also be considered as appropriate PCR reference genes. Based on the CV values of eight *S. scitamineum* genes, the expression of *S4*, *S9*, *S10*, and *S11* is less variable, whereas that of *S6* is the most variable. Based on the results using geNorm, NormFinder, and deltaCt method, *S10* and *S11* are more transcriptionally stable than *S4* and *S9*. However, the results of CV value analysis and geNorm, NormFinder, and deltaCt all indicated that *S6* is the most transcriptionally unstable gene. The *S10* gene encodes inosine-5-monophosphate dehydrogenase, which is a rate-limiting enzyme that regulates intracellular nucleic acids levels in the guanine de novo synthesis pathway [61, 62]. The *S11* gene encodes one of the key components of the signal recognition complex [63]. These features suggest that both *S10* and *S11* are indispensable proteins in living cells and thus are considered as housekeeping genes. In the same samples, the expression of *SsCMU* under the normalization of *S4* and *S6* were significantly higher (*p* < 0.05) than that of *S10*, *S11*, and their combination, indicating that the unsuitable reference gene may generate unreliable transcript profiles of the target gene. The results of the present study have proven that *S10*, *S11*, or their combination may be utilized as a reliable tool for normalization of expression data on *S. scitamineum* genes in the sugarcane buds.

## Conclusions

In this study, 10 sugarcane genes and eight *S. scitamineum* genes were selected as the candidate PCR reference genes based on the reported sugarcane transcriptome during *S. scitamineum* infection and the sugarcane-*S. scitamineum* expression microarray data. To identify the stability and the best combination and applicability of the 18 candidate PCR reference genes, they were quantitatively analyzed in sugarcane buds of different varieties and infection times by qRT-PCR. *ACAD*, *SARMp1* and “*ACAD + SARMp1*” were identified as optimal sugarcane reference genes/gene combination, and *S10, S11*, and “*S10 + S11*” were identified as optimal *S. scitamineum* reference genes/gene combination. This study facilitates gene expression analysis in sugarcane-*S. scitamineum* interaction systems.

## Additional files

Additional file 1: **Figure S1.** Melting curves of sugarcane candidate PCR reference genes. (TIF 3140 kb)

Additional file 2: **Figure S2.** Melting curves of *S. scitamineum* candidate PCR reference genes. (TIF 10506 kb)

## Abbreviations

*ACAD*: Acyl-CoA dehydrogenase
*CAC*: Clathrin adaptor complex
*CK1δ*: Casein kinase I isoform delta-like
CRG: Candidate PCR reference genes
Ct: Cycle threshold
*CUL*: Cullin
CV: Covariation
*eEF1A*: Eukaryotic elongation factor 1A
*GAPDH*: Glyceraldehyde-3-phosphate dehydrogenase
*OPR7*: 12-Oxophytodienoate reductase 7
*OTU5*: OTU domain-containing protein 5
*PABP8*: Polyadenylate-binding protein 8
qRT-PCR: Real-time quantitative PCR
RPA: Ribonuclease protection assay
RPKM: Reads per kilo bases per million reads
*S. scitamineum*: *Sporisorium scitamineum*
*SARMp1*: Serine/arginine repetitive matrix protein 1 gene
*ScChi I-3*: Sugarcane chitinase I-3
SD: Standard deviation
semi-qPCR: Semi-quantitative PCR
*SsCMU*: *S. scitamineum* chorismate mutase
SV: Stability value

## References

[1] Manners JM (2011). Functional genomics of sugarcane. Adv Bot Res.

[2] Vieira A, Talhinhas P, Loureiro A, Duplessis S, Fernandez D, Silva MC, Paulo OS, Azinheira HG (2011). Validation of RT-qPCR reference genes for in planta expression studies in *Hemileia vastatrix*, the causal agent of coffee leaf rust. Fungal Biol.

[3] Que Y, Su Y, Guo J, Wu Q, Xu L (2014). A global view of transcriptome dynamics during *Sporisorium scitamineum* challenge in sugarcane by RNA-Seq. PLoS One.

[4] Wu Q, Xu L, Guo J, Su Y, Que Y (2013). Transcriptome profile analysis of sugarcane responses to *Sporisorium scitaminea* infection using Solexa sequencing technology. Biomed Res Int.

[5] Kumar A, Naik GK, Giridhar P (2017). Dataset on exogenous application of salicylic acid and methyljasmonate and the accumulation of caffeine in young leaf tissues and catabolically inactive endosperms. Data Brief.

[6] Fan W, Chang W, Liu X, Xiao C, Yang J, Zhang Z (2017). Identification of up-regulated genes provides integrated insight into salt-induced tolerance mechanisms in *Sesuvium portulacastrum* roots. Acta Physiol Plant.

[7] Pfaffl MW (2006). Relative quantification. Real-time PCR.

[8] Silver N, Best S, Jiang J, Thein SL (2006). Selection of housekeeping genes for gene expression studies in human reticulocytes using real-time PCR. BMC Mol Biol.

[9] Thellin O, Zorzi W, Lakaye B, De Borman B, Coumans B, Hennen G, Grisar T, Igout A, Heinen E (1999). Housekeeping genes as internal standards: use and limits. J Biotechnol.

[10] Gutierrez L, Mauriat M, Pelloux J, Bellini C, van Wuytswinkel O (2008). Towards a systematic validation of references in real-time RT-PCR. Plant Cell.

[11] Sun Z-B, Li S-D, Sun M-H (2015). Selection of reliable reference genes for gene expression studies in *Clonostachys rosea* 67-1 under sclerotial induction. J Microbiol Meth.

[12] Chen C, Xie T, Ye S, Jensen AB, Eilenberg J (2016). Selection of reference genes for expression analysis in the entomophthoralean fungus *Pandora neoaphidis*. Braz J Microbiol.

[13] Volkov RA, Panchuk II, Schoffl F (2003). Heat-stress-dependency and developmental modulation of gene expression: the potential of house-keeping genes as internal standards in mRNA expression profiling using real-time RT-PCR. J Exp Bot.

[14] Nicot N, Hausman JF, Hoffmann L, Evers D (2005). Housekeeping gene selection for real-time RT-PCR normalization in potato during biotic and abiotic stress. J Exp Bot.

[15] Lilly S, Drummond R, Pearson M, MacDiarmid R (2011). Identification and validation of reference genes for normalization of transcripts from virus-infected *Arabidopsis thaliana*. Mol Plant-Microbe Interact.

[16] Jain M, Nijhawan A, Tyagi AK, Khurana JP (2006). Validation of housekeeping genes as internal control for studying gene expression in rice by quantitative real-time PCR. Biochem Biophys Res Commun.

[17] Manoli A, Sturaro A, Trevisan S, Quaggiotti S, Nonis A (2012). Evaluation of candidate reference genes for qPCR in maize. J Plant Physiol.

[18] Chandna R, Augustine R, Bisht NC (2012). Evaluation of candidate reference genes for gene expression normalization in *Brassica juncea* using real time quantitative RT-PCR. PLoS One.

[19] Paolacci AR, Tanzarella OA, Porceddu E, Ciaffi M (2009). Identification and validation of reference genes for quantitative RT-PCR normalization in wheat. BMC Mol Biol.

[20] Cortleven A, Remans T, Brenner WG, Valcke R (2009). Selection of plastid- and nuclear-encoded reference genes to study the effect of altered endogenous cytokinin content on photosynthesis genes in *Nicotiana tabacum*. Photosynth Res.

[21] Mehnaz S (2013). Microbes - friends and foes of sugarcane. J Basic Microb.

[22] Iskandar HM, Simpson RS, Casu RE, Bonnett GD, Maclean DJ, Manners JM (2004). Comparison of reference genes for quantitative real-time polymerase chain reaction analysis of gene expression. Plant Mol Biol Rep.

[23] Ling H, Wu Q, Guo J, Xu L, Que Y (2014). Comprehensive selection of reference genes for gene expression normalization in sugarcane by real time quantitative RT-PCR. PLoS One.

[24] Guo J, Ling H, Wu Q, Xu L, Que Y (2014). The choice of reference genes for assessing gene expression in sugarcane under salinity and drought stresses. Sci Rep.

[25] de Oliveira Silva RL, Silva MD, Costa Ferreira Neto JR, de Nardi CH, Chabregas SM, Burnquist WL, Kahl G, Benko-Iseppon AM, Kido EA. Validation of novel reference genes for reverse transcription quantitative real-time PCR in drought-stressed sugarcane. Scientific World J. 2014;12:357052. 10.1155/2014/357052 PMC4060590 24987730

[26] Vandesompele J, De Preter K, Pattyn F, Poppe B, Van Roy N, De Paepe A, Speleman F. Accurate normalization of real-time quantitative RT-PCR data by geometric averaging of multiple internal control genes. Genome Biol. 2002;3 research0034.1. 10.1186/gb-2002-3-7-research0034 PMC126239 12184808

[27] Andersen CL, Jensen JL, Orntoft TF (2004). Normalization of real-time quantitative reverse transcription-PCR data: a model-based variance estimation approach to identify genes suited for normalization, applied to bladder and colon cancer data sets. Cancer Res.

[28] Pfaffl MW, Tichopad A, Prgomet C, Neuvians TP (2004). Determination of stable housekeeping genes, differentially regulated target genes and sample integrity: BestKeeper - excel-based tool using pair-wise correlations. Biotechnol Lett.

[29] He Y, Yan H, Hua W, Huang Y, Wang Z (2016). Selection and validation of reference genes for quantitative real-time PCR in *gentiana macrophylla*. Front Plant Sci.

[30] Reddy DS, Bhatnagar-Mathur P, Reddy PS, Cindhuri KS, Ganesh AS, Sharma KK (2016). Identification and validation of reference genes and their impact on normalized gene expression studies across cultivated and wild *cicer* species. PLoS One.

[31] Borges AF, Fonseca C, Ferreira RB, Lourenco AM, Monteiro S (2014). Reference gene validation for quantitative RT-PCR during biotic and abiotic stresses in *vitis vinifera*. PLoS One.

[32] Machado RD, Christoff AP, Loss-Morais G, Margis-Pinheiro M, Margis R, Koerbes AP (2015). Comprehensive selection of reference genes for quantitative gene expression analysis during seed development in *Brassica napus*. Plant Cell Rep.

[33] Huang D-L, Gao Y-J, Gui Y-Y, Chen Z-L, Qin C-X, Wang M, Liao Q, Yang L-T, Li Y-R (2016). Transcriptome of high-sucrose sugarcane variety GT35. Sugar Tech.

[34] Vicentini R, Felix JD, Dornelas MC, Menossi M (2009). Characterization of a sugarcane (*Saccharum* spp.) gene homolog to the brassinosteroid insensitive1-associated receptor kinase 1 that is associated to sugar content. Plant Cell Rep.

[35] Que Y, Xu L, Wu Q, Liu Y, Ling H, Liu Y, Zhang Y, Guo J, Su Y, Chen J (2014). Genome sequencing of *Sporisorium scitamineum* provides insights into the pathogenic mechanisms of sugarcane smut. BMC Genomics.

[36] Taniguti LM, Schaker PDC, Benevenuto J, Peters LP, Carvalho G, Palhares A, Quecine MC, Nunes FRS, Kmit MCP, Wai A (2015). Complete genome sequence of *Sporisorium scitamineum* and biotrophic interaction transcriptome with sugarcane. PLoS One.

[37] Dutheil JY, Mannhaupt G, Schweizer G, Sieber CMK, Munsterkoetter M, Gueldener U, Schirawski J, Kahmann R (2016). A tale of genome compartmentalization: the evolution of virulence clusters in smut fungi. Genome Biol Evol.

[38] Yan M, Dai W, Cai E, Deng YZ, Chang C, Jiang Z, Zhang L-H (2016). Transcriptome analysis of *Sporisorium scitamineum* reveals critical environmental signals for fungal sexual mating and filamentous growth. BMC Genomics.

[39] Heinze B, Thokoane L, Williams N, Barnes J, Rutherford R (2001). The smut-sugarcane interaction as a model system for the integration of marker discovery and gene isolation. Proc S Afr Sug Technol Ass.

[40] Butterfield M, Rutherford R, Carson D, Huckett B, Bornman C (2004). Application of gene discovery to varietal improvement in sugarcane. S Afr J Bot.

[41] Que Y-X, Lin J-W, Song X-X, Xu L-P, Chen R-K (2011). Differential gene expression in sugarcane in response to challenge by fungal pathogen *Ustilago scitaminea* revealed by cDNA-AFLP. Biomed Res Int.

[42] Casu RE, Selivanova A, Perroux JM (2012). High-throughput assessment of transgene copy number in sugarcane using real-time quantitative PCR. Plant Cell Rep.

[43] Su Y, Xu L, Wang S, Wang Z, Yang Y, Chen Y, Que Y (2015). Identification, phylogeny, and transcript of chitinase family genes in sugarcane. Sci Rep.

[44] Barnabas EL, Ashwin N, Kaverinathan K, Trentin AR, Pivato M, Sundar AR, Malathi P, Viswanathan R, Rosana OB, Neethukrishna K (2016). Proteomic analysis of a compatible interaction between sugarcane and *Sporisorium scitamineum*. Proteomics.

[45] Pfaffl MW. A new mathematical model for relative quantification in real-time RT-PCR. Nucleic Acids Res. 2001;29:2002–7. 10.1093/nar/29.9.e45 PMC55695 11328886

[46] Bustin SA, Benes V, Garson JA, Hellemans J, Huggett J, Kubista M, Mueller R, Nolan T, Pfaffl MW, Shipley GL (2009). The MIQE guidelines: minimum information for publication of quantitative real-time PCR experiments. Clin Chem.

[47] Kwan Y-M, Meon S, Ho C-L, Wong M-Y (2016). Selection of reference genes for quantitative real-time PCR normalization in Ganoderma-infected oil palm (*Elaeis guineensis*) seedlings. Australas Plant Path.

[48] Vieira A, Cabral A, Fino J, Azinheira HG, Loureiro A, Talhinhas P, Pires AS, Varzea V, Moncada P, Oliveira H (2016). Comparative validation of conventional and rna-seq data-derived reference genes for qpcr expression studies of *colletotrichum kahawae*. PLoS One.

[49] Kozera B, Rapacz M (2013). Reference genes in real-time PCR. J Appl Genet.

[50] Wan H, Zhao Z, Qian C, Sui Y, Malik AA, Chen J (2010). Selection of appropriate reference genes for gene expression studies by quantitative real-time polymerase chain reaction in cucumber. Anal Biochem.

[51] Liu D, Shi L, Han C, Yu J, Li D, Zhang Y. Validation of reference genes for gene expression studies in virus-infected *Nicotiana benthamiana* using quantitative real-time PCR. PLoS One. 2012;7:e46451. 10.1371/journal.pone.0046451 PMC3460881 23029521

[52] Chi C, Shen Y, Yin L, Ke X, Han D, Zuo Y (2016). Selection and validation of reference genes for gene expression analysis in *Vigna angularis* using quantitative real-time RT-PCR. PLoS One.

[53] Singh V, Kaul SC, Wadhwa R, Pati PK (2015). Evaluation and selection of candidate reference genes for normalization of quantitative RT-PCR in *Withania somnifera* (L.) Dunal. PLoS One.

[54] Xiao D, Zhang N-W, Zhao J-J, Bonnema G, Hou X-L (2012). Validation of reference genes for real-time quantitative PCR normalisation in non-heading Chinese cabbage. Funct Plant Biol.

[55] Camoes F, Islinger M, Guimaraes SC, Kilaru S, Schuster M, Godinho LF, Steinberg G, Schrader M (2015). New insights into the peroxisomal protein inventory: acyl-CoA oxidases and -dehydrogenases are an ancient feature of peroxisomes. BBA-Mol Cell Res.

[56] Palusa SG, Reddy ASN (2010). Extensive coupling of alternative splicing of pre-mRNAs of serine/arginine (SR) genes with nonsense-mediated decay. New Phytol.

[57] Reddy ASN, Ali GS (2011). Plant serine/arginine-rich proteins: roles in precursor messenger RNA splicing, plant development, and stress responses. Wires RNA.

[58] Hu Y, Chen H, Luo C, Dong L, Zhang S, He X, Huang G (2014). Selection of reference genes for real-time quantitative PCR studies of kumquat in various tissues and under abiotic stress. Sci Hortic-Amsterdam.

[59] Ribeiro PR, Dekkers BJW, Fernandez LG, de Castro RD, Ligterink W, Hilhorst HWM (2014). Identification of reference genes for gene expression studies during seed germination and seedling establishment in *Ricinus communis* L. Seed Sci Res.

[60] Ye X, Zhang F, Tao Y, Song S, Fang J (2015). Reference gene selection for quantitative real-time PCR normalization in different cherry genotypes, developmental stages and organs. Sci Hortic-Amsterdam..

[61] Buey RM, Ledesma-Amaro R, Balsera M, Maria de Pereda J, Luis Revuelta J (2015). Increased riboflavin production by manipulation of inosine 5′-monophosphate dehydrogenase in *Ashbya gossypii*. Appl Microbiol Biotechnol.

[62] Park J-H, Ahn SH (2010). IMP dehydrogenase is recruited to the transcription complex through serine 2 phosphorylation of RNA polymerase II. Biochem Biophys Res Commun.

[63] Hann BC, Stirling CJ, Walter P (1992). *SEC65* gene product is a subunit of the yeast signal recognition particle required for its integrity. Nature.

