# Supplementary figures and images for: Identification and evaluation of PCR reference genes for host and pathogen in sugarcane-Sporisorium scitamineum interaction system
Source: BMC Genomics. 2018 Jun 19;19:479. doi: 10.1186/s12864-018-4854-z (PMC6006842; doi:10.1186/s12864-018-4854-z)

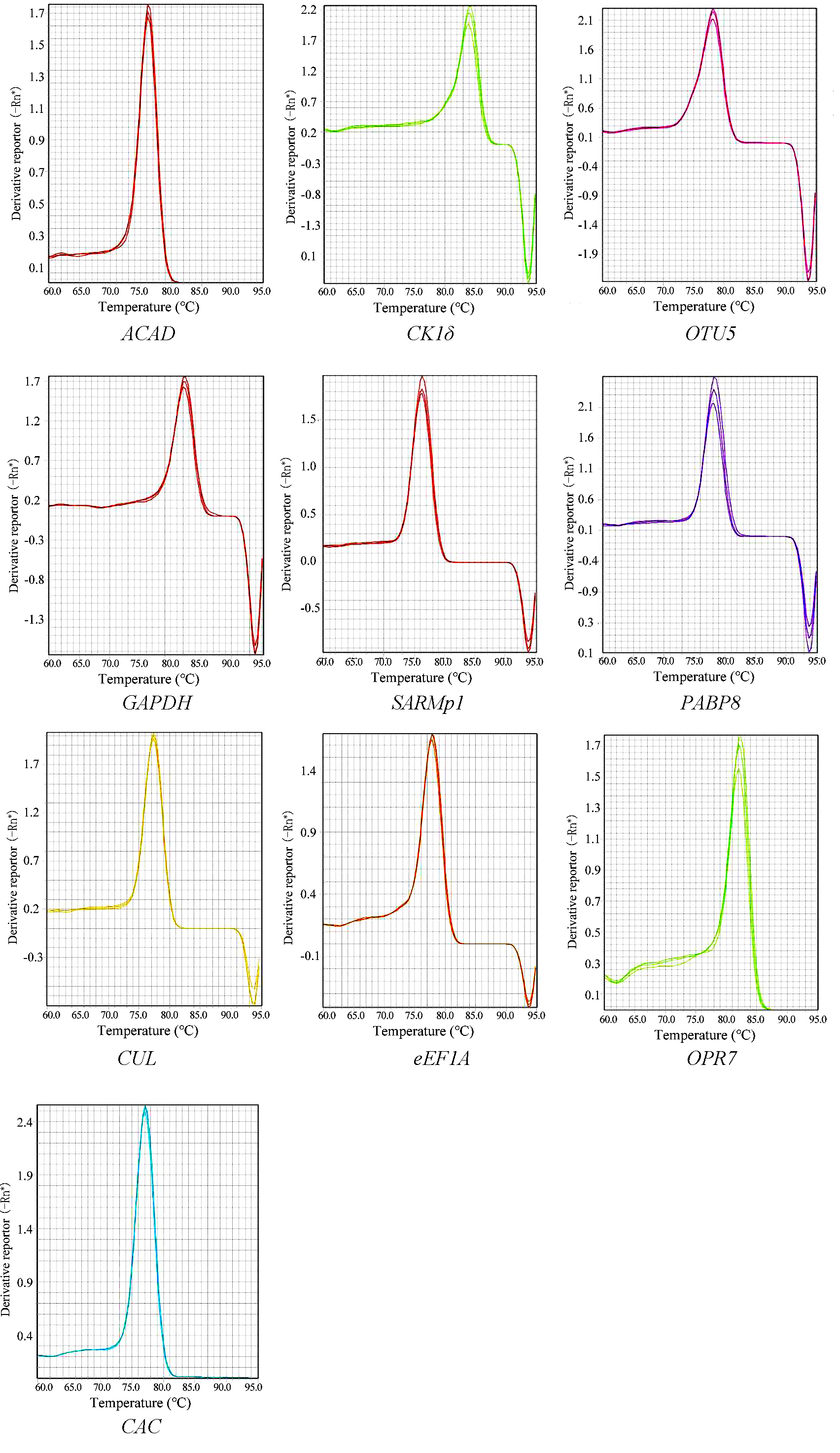

Supplement: Supplementary file 1 — Figure S1. Melting curves of sugarcane candidate PCR reference genes. (TIF 3140 kb) [file 12864_2018_4854_MOESM1_ESM.tif]

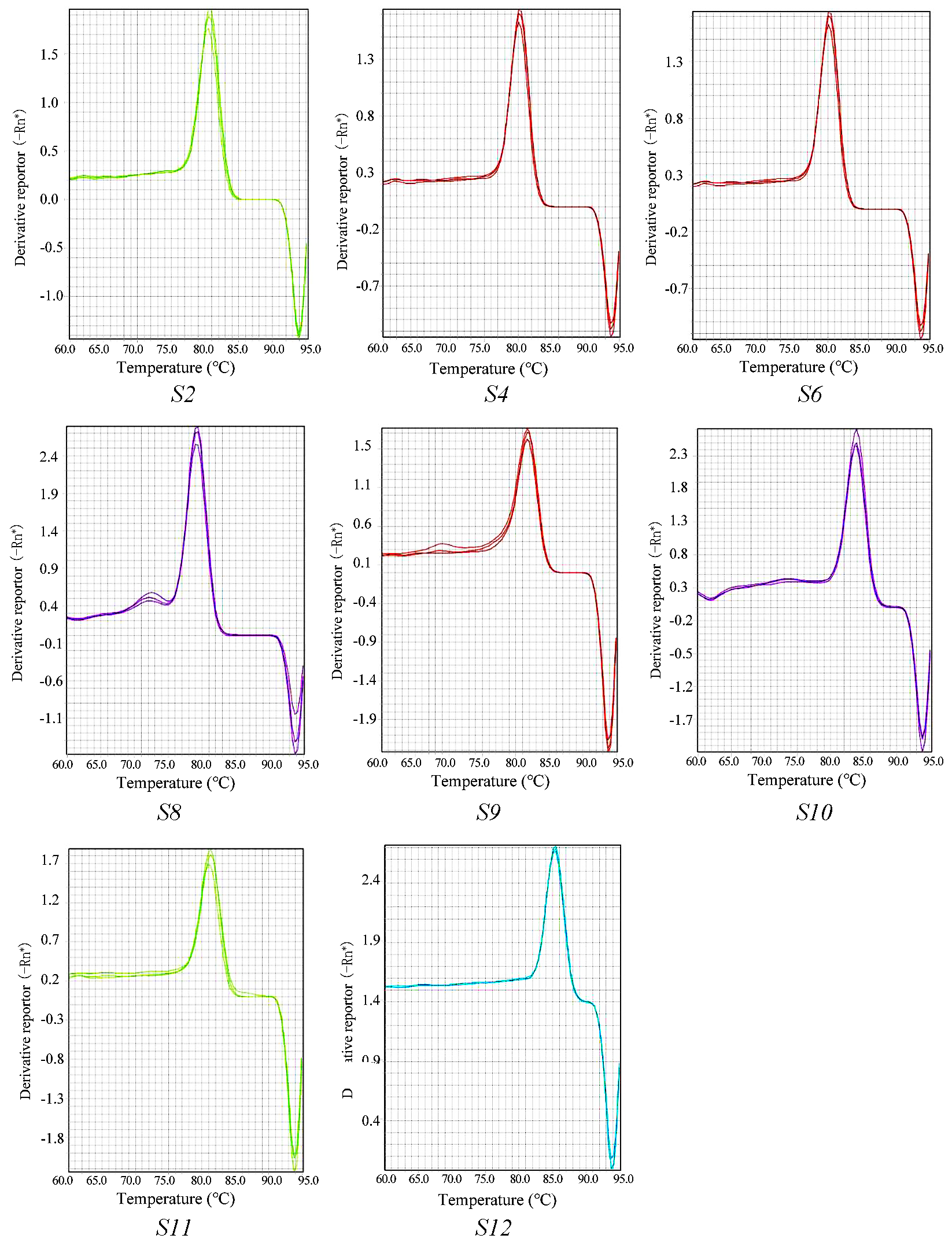

Supplement: Supplementary file 2 — Figure S2. Melting curves of S. scitamineum candidate PCR reference genes. (TIF 10506 kb) [file 12864_2018_4854_MOESM2_ESM.tif]
